# Delayed Complications After Maxillary Sinus Floor Augmentation with a Bovine Xenohybrid Bone Graft: A Retrospective Clinical Case Series

**DOI:** 10.3390/diagnostics16193098

**Published:** 2026-09-24

**Authors:** Patrick Bandura, Christian Philipp Schnell, Claas Willem Molsen, Anna Sophia Bandura, Pascal Grün, Marius Meier, Maximilian Jung, Flora Turhani, Sebastian Fitzek, Dritan Turhani

**Affiliations:** 1 Center for Oral and Maxillofacial Surgery, Department of Dentistry, Faculty of Medicine and Dentistry, Danube Private University, Steiner Landstrasse 124, A-3500 Krems-Stein, Austria; patrick.bandura@dp-uni.ac.at (P.B.); schnell.christian@dp-uni.eu (C.P.S.); molsen.claas@dp-uni.eu (C.W.M.); annasophia.bandura@dp-uni.ac.at (A.S.B.); pascal.gruen@dp-uni.ac.at (P.G.); meier_marius_1@hotmail.com (M.M.); jung.maximilian@dp-uni.eu (M.J.); turhani.flora@dp-uni.eu (F.T.); 2 Clinical Application of Artificial Intelligence in Dentistry (CAAID) Group, Department of Dentistry, Faculty of Medicine and Dentistry, Danube Private University, Steiner Landstrasse 124, A-3500 Krems-Stein, Austria; 3 Dental Practice, 9490 Vaduz, Liechtenstein; 4 Medical Image Analysis & Artificial Intelligence (MIAAI) Group, Faculty of Medicine and Dentistry, Danube Private University, Steiner Landstrasse 124, 3500 Krems An Der Donau, Austria; sebastian.fitzek@dp-uni.ac.at

**Keywords:** maxillary sinus floor augmentation, sinus floor elevation, xenohybrid bone graft, SmartBone, late complications, graft failure, dental implants, case series

## Abstract

**Background/Objectives:** Maxillary sinus floor augmentation (MSFA) via the lateral approach is a well-established procedure for prosthetic rehabilitation of atrophic posterior maxillae. However, late complications are often nonspecific, with an incompletely understood aetiology. We evaluated patients with atypical late complications post-MSFA with bovine xenohybrid bone substitute NaturesQue SemOss B (BEGO GmbH, Bremen, Germany), describing their presentation, management, and outcomes. **Methods:** This retrospective, complication-selected clinical case series examined 47 patients who underwent lateral window MSFA with this graft from September 2020 to March 2023 in a single university department. Twelve complication cases were identified during the study period. Atypical late complications were defined as graft-associated clinical and/or radiological adverse outcomes, excluding peri-implantitis, that first manifested > 6 mo postoperatively. Baseline characteristics, perioperative findings, conservative and surgical management (including graft removal and reaugmentation), histology, and follow-up were assessed. **Results:** Twelve complication cases were identified among 47 patients treated during the study period. Seven experienced implant loss; seven, partial or complete lack of graft osseointegration; six, pain; and seven, signs of sinus infection. Histological analysis (6/12) revealed lymphocytes, plasma cells, and fibroblastic changes without detectable macrophages. No intraoperative complications were observed. **Conclusions:** Atypical late complications affected 25.5% of the patients. Despite comprehensive clinical, radiological, and histological assessments, the aetiology remained unclear, and the findings did not indicate a peri-implantitis-type infection; we hypothesised impaired early phase wound healing These patients were amenable to repeat augmentation post-non-osseointegrated graft removal, although larger studies are required.

## 1. Introduction

Maxillary sinus floor augmentation (MSFA) via the lateral approach is a reliable and well-established procedure for the rehabilitation of patients with partial or total tooth loss [1,2,3]. In cases of pronounced atrophy and maxillary sinus pneumatization, vertical bone height enables the placement of fixed dental prostheses, strategic addition of abutments, and improved stability of removable prosthetic restorations. Numerous surgical approaches have been shown to achieve excellent implant survival rates, employing autogenous bone, bone-substitute materials, combinations of both, or no grafting materials [4,5,6,7].

However, since MSFA is technique-sensitive, numerous complications and adverse effects have been reported with varying and contradictory effects on treatment success. Typical and widely prevalent intraoperative or near postoperative complications include perioperative or postoperative haemorrhage, local wound dehiscence, and graft displacement with consecutive acute sinus inflammation [8,9,10]. The most prevalent complication is Schneiderian membrane perforation, whose multifactorial aetiology can be categorised into anatomical, patient-related, and technical factors [11]. Furthermore, complications including neurological damage (temporary or permanent palatal numbness), implant displacement, oroantral fistula, poor graft adaptation, and benign paroxysmal positional vertigo [12] (see also Valentini & Stacchi 2024 [13]), among others, should be acknowledged. This contrasts with the multitude of late complications, such as late sinus graft infection, loss of osseointegration, implant displacement, cyst formation, and chronic maxillary sinusitis [10,14,15,16].

In contrast to intraoperative and early postoperative complications, a considerable proportion of late complications are characterised by the fact that their pathogenesis can only be partially explained, and their aetiology frequently remains incompletely delineated. Late complications may go unrecognised for a prolonged period, as the presenting symptoms are frequently nonspecific and non-acute, or the condition may remain completely asymptomatic until disease progression leads to exacerbation or onset of a secondary complication. In addition to efforts to employ a standardised approach, the management of late complications frequently entails deviations from the planned course of treatment due to the individual circumstances of the patient.

In a recent study, atypical late complications that occurred independently of peri-implantitis were described, suggesting a possible association with graft material properties [17].

From September 2020 to March 2023, MSFA was performed in a cohort of 47 patients at the Department of Oral and Maxillofacial Surgery, Danube Private University (DPU), Krems-Stein, Austria. Grün et al. [18] reported that a considerable proportion of such patients subsequently exhibited atypical late complications. The cases in our study, originally from our previous paper [18], involved the use of the xenogeneic bovine bone substitute material naturesQue SemOss B (BEGO GmbH, Bremen, Germany), also marketed as SmartBone^®^ (Industrie Biomediche Insubri SA, Mezzovico-Vira, Switzerland). The material comprises a deproteinised bovine cancellous bone matrix coated with a poly(L-lactide)/poly(ε-caprolactone) copolymer containing incorporated collagen-derived fragments. This article focuses on investigating late complication aetiology and analysing these cases.

The bone substitute was structured as a hybrid scaffold based on the deproteinised bovine bone. Aliphatic polyesters and their copolymers, including poly(lactic acid) and poly(gamma-caprolactone), are characterised by their hydrolytic degradation properties. These materials exhibit good biocompatibility and the capacity to undergo biodegradation, resulting in nontoxic physiological byproducts. The combination of these polymers improves their mechanical properties in terms of strength and tenacity [19,20]. The incorporation of gelatin was intended to improve the overall hydrophilicity with good wettability [21]. The amalgamation of materials fosters a conducive environment for blood clot formation through fibrinogen adsorption and platelet adhesion as well as for cell adhesion and cell growth [21,22]. In addition to orthopaedic applications, the material has been approved for use in regenerative and alveolar ridge augmentation techniques as well as sinus floor elevation procedures in dentistry. The increased number of long-term complications observed in cases in which this material has been used in MSFA raises a clinically significant question.

The objective of this retrospective, complication-selected clinical case series was to provide a comprehensive evaluation of patients who underwent MSFA using the augmentation material SmartBone^®^ during the surgical rehabilitation process. The primary focus of this study was on atypical late complications that may arise following this procedure and the available therapeutic approaches for their management. The analysis was conducted by considering the patients’ medical histories and prior dental treatments. This case series is reported in line with the PROCESS guidelines wherever possible [23].

## 2. Materials and Methods

### 2.1. Participants and Case Selections

The patients included in this retrospective case series were treated at the Department of Oral and Maxillofacial Surgery of DPU, Krems-Stein, Austria, originally from our previous study [18]. This retrospective case series examined patients who underwent lateral MSFA (7 times left side, 5 times right side) using bovine xenohybrid bone grafts (SmartBone^®^) and subsequently developed atypical late complications. The study was deliberately conducted at the patient level because, with one exception, the surgeries were performed on one side only. Furthermore, in the case of the bilateral procedure, there was a significant time interval between the surgeries. This definition encompassed all cases of clinical and/or radiological adverse outcomes associated with the augmentation material that first manifested > 6 mo after primary sinus floor elevation and either persisted over time or required surgical intervention. This categorically precluded clinical manifestations of peri-implantitis. The inclusion criteria further stipulated that the patients were of adult age (≥18 y) and that complete perioperative documentation, including radiographic imaging, medical history, and follow-up data, were available. Treatments within the cohort that did not exhibit any atypical late complications at the time of manuscript preparation (7 June 2026) were also excluded therefore the analytical dataset was complication-selected. A total of 12 patients were observed. The study was conducted in accordance with the Declaration of Helsinki and was approved by the Central Ethical Review Board of the Federal State of Lower Austria (approval number: GZ: DPU-EK/069). Informed Consent Statement: Informed consent was obtained from all the participants involved in the study. Written informed consent was obtained from the patients for the publication of this paper.

### 2.2. Recruitment

The study population comprised patients from the University Hospital and those referred to the Department of Oral and Maxillofacial Surgery. Following a detailed evaluation of the patient’s medical history, comprehensive diagnostic evaluation, and patient counselling, the indications for treatment were assessed. Informed consent was obtained from the patients prior to the procedure. In this context, the patient was provided with a benefit-risk assessment and a detailed explanation of the potential risks and complications associated with the procedure, and alternative treatment modalities were discussed. The patient was granted sufficient time to deliberate on the available options. No incentives were offered to participate or remain in the study.

### 2.3. Pre-Intervention Patient Optimisation

The patients underwent a standardised preoperative protocol to minimise the occurrence of complications and optimise treatment outcomes. In addition to a detailed medical history encompassing both general medical and specific dental aspects, as well as a family medical history, cone-beam computed tomography (CBCT) and blood tests were requested to complement the comprehensive clinical diagnosis. The complete blood count without differential included leucocytes, erythrocytes, platelet count, haemoglobin concentration, haematocrit, red blood cell distribution width, mean corpuscular haemoglobin, mean corpuscular volume, mean corpuscular haemoglobin concentration, and mean platelet volume. Vitamin D levels were measured in all the patients. Furthermore, glycated haemoglobin levels were measured in the patients diagnosed with diabetes mellitus, whereas the International Normalised Ratio was recorded in patients undergoing anticoagulant therapy with a history of coumarin derivative use. Preoperative oral hygiene procedures in conjunction with individualised patient instructions on enhancing oral hygiene practices are mandatory. In the event of any suspicion of an untreated or inadequately treated preexisting condition, or if the condition was in a progressive stage, the surgical procedure was postponed. At least 1 wk prior to the procedure, the patients were provided with a comprehensive and personalised information sheet to take home, and their medical history was reviewed to ensure its currency.

### 2.4. Interventions and Operator Details

The primary procedure evaluated was lateral window MSFA using a bovine xenohybrid bone graft material (natureQue SemOss B). This approach was applied in cases of insufficient bone volume, defined as a residual bone height of ≤5 mm, with the aim of enabling subsequent implant-supported prosthetic rehabilitation. The surgical interventions were performed by the same experienced maxillofacial surgeon at the University Department of Oral and Maxillofacial Surgery.

The patients were administered 40 mg of methylprednisolone (Fidia Pharma GmbH, Monheim am Rhine, Germany) 30–60 min pre-procedure and again the following day to prevent swelling. Surgical procedures were performed under sterile conditions. Under local anaesthesia, a mucoperiosteal flap was prepared and mobilised to expose the lateral sinus wall. To create a window approximately 3 mm above the sinus floor, a round stainless-steel burr was initially employed, followed by a round diamond burr. The membrane was then meticulously mobilised, and its integrity was examined both visually and through the Valsalva manoeuvre. The resulting space was filled with 0.5–2 g of bone graft material depending on the planned ongoing treatment. The material was carefully injected using Freer so that the resulting cavity was completely filled but did not condense. When this was feasible, the implant was simultaneously inserted. The implants placed during the same surgical appointment (single-stage procedure) were the Bego S-Line (BEGO GmbH) and Biomet 3i (ZimVie, Westminster, CO, USA). A resorbable collagen membrane (TachoSil^®^, Corza Medical, Roggwil, Switzerland) was placed over the access site, and the mucoperiosteal flap was repositioned. Final primary wound closure was performed using 5.0 nonabsorbable polypropylene sutures to ensure tension-free conditions. The patient was provided with comprehensive postoperative counsel and directed to administer 875 mg/125 mg of amoxicillin/clavulanic acid (Augmentin^®^, GlaxoSmithKline, London, UK) twice daily for a duration of 5 d. In cases of penicillin allergy, clindamycin (Dalacin CTM 300 mg, Pfizer, New York, NY, USA) was prescribed thrice daily for 5 d.

In the event that implants are placed in a second, separate session (two-stage procedure), it is imperative to consider a healing period of at least 6 mo into account. The following materials were used: Bego S-Line, Biomet 3i, and Straumann BLX SL-active (Institut Straumann AG, Basel, Switzerland).

Treatment measures implemented after the initial onset and subsequent occurrence of late complications were guided by the patient’s individual symptoms, clinical presentation, level of compliance, and available treatment options for corrective action. The clinical decision-making process was undertaken in consultation with the patient and informed by a detailed assessment of pain history, comprehensive clinical examination, and radiological follow-up.

Where indicated, secondary surgery was performed to re-establish lateral access to the maxillary sinus, with complete removal of the non-osseointegrated graft material and any affected implants (same precautions; see above). Furthermore, the affected regions of the maxillary sinuses were irrigated with an antiseptic solution consisting of polyvidone-iodine (Betaisodona^®^, Taw Pharma, Dublin, Ireland) diluted 1:1 with sterile saline solution. The extracted substances comprising the graft and surrounding tissues were subjected to histological analysis (hematoxilin-eosin method, paraffin fixation, slices of 20 × 20 × 3 mm under 10 µm). Nor was a microbiological culture, PCR, Gram staining, PAS/GMS, or other bacterial/fungal methods performed, neither was the pathologist blinded to clinical information.

Following extended consultation with the patient and considering every foreseeable risk factor as well as the patient’s current general state of health and dental condition, MSFA was re-performed in 3 of the patients. The essential prerequisites for this were the complete absence of symptoms, unremarkable clinical and radiological findings, and a period of at least 6 mo following the removal of all non-osseointegrated materials and any affected implants. The surgical procedure was performed with the same cautionary measures that were implemented during the preliminary procedure (see above), with the augmentation material Endobon^®^ Xenograft (ZimVie) being utilised.

### 2.5. Postoperative Care and Follow-Up

In addition to perioperative antibiotic medication, postoperative pain relief was facilitated by the administration of Parkemed^®^ (Pfizer, New York, NY, USA). Otrivin^®^ (Haleon Group of Companies, Weybridge, Surrey, UK) was prescribed for a limited period in order to reduce swelling of the respiratory epithelium and chamomile extract (Kamillosan^®^, Cooper Consumer Health SAS, Paris, France), to support daily oral hygiene while minimising tissue irritation. Although this should not be interpreted as an excluding factor for an infection. Preoperative and postoperative systemic medication could modify inflammatory and histological findings. CBCT was performed immediately postoperatively to assess the spatial positioning of the bone graft and inserted implants. The first follow-up examination was performed within the first 2 d postoperatively, and the sutures were removed after 14 d.

The clinical and radiological evaluations were repeated at regular intervals of up to 6 mo, with consideration given to any anamnesis, pain, or other discomforts described by the patient; gingival, mucosal, and peri-implant mucosal tissue conspicuities; presence of sinus tracts; or radiographic alterations of the maxillary sinus, swollen membrane (>1 mm), bone graft material, and implants and surrounding tissues. An annual CBCT scan was performed.

Subsequent evaluations were conducted by a university professor or senior physician from the same Department of Oral and Maxillofacial Surgery.

### 2.6. Outcome Variable and Covariates

The primary outcome of interest was to investigate the symptoms, clinical and radiological features, and initial manifestations of atypical late complications following lateral-approach MSFA and their developmental trajectory following conservative or surgical intervention, including repeated sinus lift augmentation and further follow-up care. Consequently, the patients were designated as the primary unit of analysis. The assessment encompassed patient age (y) and sex; a comprehensive evaluation of preexisting medical conditions and medications; the patient’s detailed dental history; preoperative procedures performed at the designated surgical site; the materials used, including the quantity of graft (mm^3^) and endosseous implant systems; timing of dental implant insertion; any intraoperative or postoperative complications that may have emerged during the primary or subsequent procedures; and the current condition of each individual patient. Therefore, sinus, implant and prosthetic functional levels were analysed and evaluated.

## 3. Results

### 3.1. Participants

Grün et al. [18] reported the identification of 9 cases of failed MSFA in 47 patients who had received treatment with the aforementioned augmentation material, (naturesQue SemOss B). A total of 84 implants were inserted (one implant/12 patients, two implants/33 patients, three implants/2 patients and only 1 patient did not receive a dental implant) from different manufacturers: Biomet 3i (ZimVie, Westminster, CO, USA): 30 T3^®^, 2 TPT^®^, 2 T3ST^®^; Neoss (Neoss, Cologne, Germany): 2 ProActive Tapered; BEGO (BEGO, Bremen, Germany): 36 S-Line, 2 Semados SC/SCX; SIC (SIC, Basel, Switzerland): 2 SiCmax Screw Implant; and Straumann (Institut Straumann AG, Basel, Switzerland): 8 BLX SL-Active. This number increased to 12, despite the fact that no further use of this material was made.

Our patient cohort comprised six male (aged 75–45 y, average age of 60.67) and six female patients (aged 67–42 y, average age of 54.83).

The patients were categorised as either Class I or Class II according to the American Society of Anesthesiologists classification system. Although comorbidities were present, none were deemed to be of acute necessity for treatment as all the patients had been treated prior to surgery. The management of chronic conditions involves a combination of monitoring and medication administration by a specialist in internal medicine. Nine patients were not taking any medications and had no comorbidities. One patient had both type 2 diabetes mellitus and hypertension. The other patient had a history of coagulopathy and hypertension. Finally, one patient exhibited latex allergy in addition to steatotic liver disease of unclear origin, thyroid dysfunction, hypertension, and craniomandibular dysfunction. Consequently, the treatment was adopted in accordance with our hypothesis. However, it should be noted that medication use does not always correlate with comorbidities. One patient also reported taking an antipsychotic drug (quetiapine) but was unable to provide further information regarding the pharmaceutical manufacturer or dosage. Another patient reported taking montelukast (leukotriene receptor antagonist), Spiriva^®^ (Boehringer Ingelheim Pharma GmbH & Co., KG, Ingelheim on the Rhine, Germany), Symbiocort forte Turbohaler^®^ (budesonide/formoterol, AstraZeneca, Cambridge, England), and Berodual^®^ (Boehringer Ingelheim Pharma GmbH & Co., KG). However, no information has been provided regarding these doses.

MSFA can be considered neither severe nor urgent. All the patients were treated under local anaesthesia. Surgeries were scheduled without the urgent need for treatment. The surgical procedure was conducted under stringent sterile conditions and included the subsequent scheduled implant recovery procedure.

The patients’ baseline characteristics are summarised in Table 1. Specifically, this encompasses age, sex, preexisting conditions, risk factors, medication use, and prior surgical procedures performed at the site of the subsequent MSFA.

### 3.2. Deviation from the Initial Management Plan and Outcome

This cohort describes only patients in whom the original treatment plan deviated owing to the circumstances described earlier. A comprehensive schematic of the treatment and procedural protocols is presented in Figure 1. The proportion of MSFA procedures associated with complications was exceptionally high at 12 out of 47. A one-stage approach was chosen for 4 of these 12 patients. In seven cases, implantation subsequently occurred at least 6 mo later, making it a two-stage approach. In only one case, complications arose prior to the planned implantation; as a result, implantation did not occur.

In the remaining patients, no symptoms were observed at the time of implantation. If complications arise after implantation, the clinician has two options for further treatment, which is not the case with conventional peri-implantitis. These consist of either removing or retaining the implants. Under these circumstances in our study, the decision to remove the implant was made based on the implant mobility, the patient’s level of discomfort, and the probability of reversal.

Following a comprehensive clinical evaluation, a decision could be made to proceed with implant removal. In each of these cases, in addition to the explanation, the portion of the bone graft that had not completely osseointegrated with the bone, granulation tissue, or inflamed connective tissue also had to be removed. However, portions of the graft that were fully osseointegrated during the surgery were left in situ.

Adequate restoration is required to ensure sufficient support in cases of implant and bone graft loss. This objective was accomplished by implementing an interim denture or in cases where residual implants were present through the adaptation of the restoration. Re-MSFA was planned for each of these patients, provided they were compliant, consented to the procedure, and were free of inflammation in the affected area. Endobone^®^ was selected for the re-MSFA procedure, and Bego S-Line, Biomet 3i, and Straumann BLX SL-Active implants were placed. In cases where the patients exhibited no mobility and only minor symptoms, implants and restorations were left in place and monitored every 6 mo. The conservative therapeutic approach encompassed a combination of antibiotics and medications to reduce mucosal swelling in cases with severe symptoms and no consent for surgery.

Following the manifestation of the initial symptoms, two patients opted to transition to a different practitioner. In both cases, however, the implants were in situ without mobility at the time of the change in practitioner. Consequently, the denominator of functional outcomes was 10 as opposed to 12.

### 3.3. Outcomes and Follow-Up

With the exception of the two patients who changed practitioners, all 10 remaining patients were still being treated at the Maxillofacial Surgery Centre of DPU and were called in for follow-ups every 6 mo.

In one case, symptoms manifested prior to implant insertion. In two cases, the implants were left in situ. In this case, the moderate symptoms manifested as a ‘dull’ sensation; however, no implant loosening was observed. In one of these cases, a fistula tract developed and healed without further complications after curetting. The fistula manifested 1 y post-implantation (Figure 1).

Another patient reported mild symptoms 4 y postoperatively in the form of swelling and a tender nasal septum. In this instance, the conservative treatment option was selected, encompassing the administration of antibiotics (Augmentin^®^) and decongestant nasal drops (Otrivin^®^).

Seven patients experienced loss of implants. The following conclusions were drawn from the cases under consideration:

In two cases, it was possible to fit a new fixed restoration using re-MSFA. In another patient, re-MSFA was performed without implantation (Figure 2).

One of these patients exhibited a yellowish hyperplastic maxillary sinus mucosa at the time of the initial explanation. In another patient, re-MSFA was performed; however, no implants have been placed at the time of writing. Subsequent follow-up revealed the development of a fistula. However, the patient also reported having blown their nose and sneezed several times. In one patient, despite the removal of both implants and a portion of the bone graft, subsequent analysis indicated the feasibility of reimplantation in the remaining osseointegrated bone graft without re-MSFA. In one case, the patient developed a persistent oroantral communication following the removal of the mobile implant, which could not be closed even after two attempts at coverage. Finally, the clinician opted for two-layer coverage using the buccal fat pad to resolve the issue. A fixed restoration is currently not planned for this patient. In two cases, a new restoration via further MSFA and implants was planned but has not yet been carried out at the time of writing.

It is noteworthy that one patient has not received any implants to date owing to the presence of subjective symptoms and hypoplastic changes in the maxillary sinus mucosa. The patient lost the bone graft material without having undergone implantation. Re-MFSA is currently being planned as the patient has been symptom-free for at least 6 mo. The initial bone graft was excised during the course of surgical treatment of the fistula tract, which was one of the three tracts observed in this cohort.

The absence of osseointegration in an implant is typically identified during implant recovery, impression taking, loading, or in the immediate aftermath. Conversely, it is more difficult to attribute subjective symptoms to specific moments. While some patients reported these shortly after the implants were recovered, in one patient, they did not appear until 3 y and 7 mo after loading. The presence of fistula tracts was observed in two cases 1 y post-MSFA and in one patient, just 3 wk after surgery on the maxillary sinus.

Table 2 summarises the patients’ clinical characteristics. The conditions cover all surgical and prosthetic procedures performed until the initial manifestation of symptoms or complications. The augmentation material used was natureQue SemOss B. Symptoms & findings summarises all discomfort reported by the patients, extraoral and intraoral findings, and any radiographic abnormalities associated with atypical late complications. Intervention provided a comprehensive overview of all the measures implemented in the aftermath of the onset of complications. The current situation describes the status of all the affected patients at the time of publication.

The standardised outcome measures were meticulously recorded and are summarised in Table 3. Functional, radiological, and clinical outcome measures were distinguished, with the latter further subdivided into intraoperative, early, and late complications. Primary MSFA and re-MSFAs were analysed separately for each patient. The two patients who transferred to different practitioners appeared to be dissatisfied with the treatment duration, and limited success was achieved. MFSA, particularly when associated with complications, can be a nerve-wracking process, with up to 18 mo sometimes passing between the initial treatment and final restoration.

### 3.4. Intervention Adherence and Compliance

Postoperative care was provided at the DPU dental clinic in accordance with a standardised protocol as mentioned in the Materials and Methods section. Postoperative care is crucial for patient recovery following maxillary sinus surgery. To ensure optimal outcomes, the patients were provided with an information guide outlining the necessary steps for postoperative behaviour. This was given immediately postoperatively, along with prescriptions for antibiotics, analgesics, and decongestant medications, as well as a cold pack. The surgeon discussed the use of these with the patient. Adaptation to the medication was performed if necessary. The guide contained standard information regarding MSFA and implantation. These included recommendations to patients to avoid smoking, consuming alcohol, strenuous overhead activities, sneezing, or blowing their nose; to practice proper cooling; and to maintain optimal oral hygiene for 7 d.

In two cases, sneezing and nasal clearance were reported. Subsequently, both patients developed healed fistulas. One of these fistulas did not occur with the initial MFSA but with re-MFSA. The remaining patients appeared to be highly compliant, as demonstrated by their consistent attendance at follow-up appointments and adherence to the prescribed medication. There was no systematic recording of adherence to daily routines; for example, regarding the duration of an existing pressure sore until the patient visited a dental clinic.

### 3.5. Complications and Adverse Outcomes

This report covers 12 patients who underwent MSFA with the aforementioned bone graft material (naturesQue SemOss B). For completeness, the following implants were used: Bego S-Line, Biomet 3i, and Straumann BLX SL-Active. We observed the following serious adverse events:

1. Pain and disorders in six cases as late complications.
2. Partial or total lack of osseointegration of the bone graft material in seven cases.
3. Implant loss in seven cases.
4. At the time of publication, due to late complications that had arisen, no prosthetic rehabilitation was performed in five cases, potentially resulting in a corresponding reduction in oral health-related quality of life.

Further, ancillary complications observed were:

1. Fistula tracts to the initial MSFA in two cases.
2. Persistent oroantral communication following explantation in one case.
3. Sinusitis symptoms or other signs of infection in seven cases as late complications.
4. Confirmed hyperplastic changes (>1 mm) in the Schneiderian membrane in two cases.

## 4. Discussion

### 4.1. Key Results

To the best of our knowledge, this is only the second study [24] to address the increased incidence of late complications following MSFA using NaturesQue SemOss B or SmartBone^®^. The elevated incidence of complications associated with MSFA compared with other established bone substitute materials underscores the pressing necessity for a comprehensive investigation of this phenomenon. Our patients, who were treated with the xenogeneic, bovine graft material under MSFA, demonstrated a markedly high observed frequency rate of non-osseointegrated graft material and implant loss, accompanied by subjective symptoms and pain, and/or signs of inflammation, which became apparent at least 6 mo after the surgical procedure.

Of the 47 patients, 7 experienced implant loss, 1 never received any implants, and 4 encountered different forms of complications. In one case, an implant was placed in the portion of the graft that had not been removed. However, this did not affect the outcomes. In two cases, re-MSFA was employed, resulting in the successful placement of a definitive restoration. In one case, the graft was inserted as part of a two-stage approach; however, no implant has been placed thus far. In all three cases, the fistula tract exhibited signs of healing, although the graft had to be excised in one case.

### 4.2. Scientific Context and Implications

The desired outcome in the context of MSFA is a loaded implant at the augmented site. According to BEGO’s instructions, the augmented site may be loaded after 6 mo at the earliest. The healing time depends on whether a one-step or two-step approach is employed. Single-stage implantation is feasible if there are more than 4 mm of residual bone height and no other factors, such as primary stability, preclude it [25]. In this case, the implant could be loaded after 6 mo. With the two-step approach, implantation can only occur after the augmentation site has healed. In this case, the patients needed to wait an additional 3–4 mo. The literature states that although xenograft materials are surrounded by bone matrix after 6 mo, the actual histological bone structure is not present until 9 mo later [26]. As has been considered for some patients, an implant in MSFA can be impressioned and loaded from 6–13 mo postoperatively.

After the publication of the same retrospective cohort study by Grün et al. [18], the rate of patients demonstrating complications increased from 19.1% to 25.5%. These complications were atypical. Therefore, further cases are expected to emerge. This observation is consistent with the findings of the study conducted by Ben-Dor et al. [24]. In this cohort, complications occurred in 22 of their 67 patients (32.8%). An examination of this xenohybrid bovine graft material in a different study revealed an immature trabecular microarchitecture at non-inflammatory sites [27]. In a study by Taschieri et al. [28], 10 patients who had undergone a sinus lift using SmartBone^®^ were examined. In the present study, a period of 1 y elapsed following definitive implant placement, at which point it was observed that all the implants had become integrated into the bone without complications. Another study found no signs of inflammation 6 mo after augmentation and good osteoconduction [20].

However, the extant research does not offer a plausible explanation for these circumstances. The findings of these studies do not allow the determination of which medication, treatment modality, or prerequisite is associated with success; therefore, clinical implications cannot be made on this basis. Claims pertaining to partial graft removal require further investigation. The same can be said of the decision between conservative and surgical treatment. The paucity of specialist literature on the management of complications following MSFA is a matter of concern. Nevertheless, a consensus study by Testori et al. [29] provided valuable guidance. For instance, experts considered it appropriate to administer antibiotics to patients with swelling and pain. Furthermore, the presence of a foul odour and taste was considered a possible complication, although this was not reported in any of the patients examined in this study. Additionally, it is important to note that swelling of the Schneider’s membrane does not necessarily indicate a failed MSFA [29].

The occurrence of atypical late complications was not consistent with the ‘gold standard’. For over a decade, post-MSFA success rates in the posterior region have been comparable to those in regular bone [3,7,30]. However, techniques and materials have been established to play a role [31,32].

The current circumstances indicate the absence of osseoconduction of the augmentation material due to the convergence of clinical, radiological, and histological findings. This process leads to encapsulation of the graft material. Histological examination of the removed bone graft confirmed this. However, evidence available to assess this topic is limited. Only one case series to date has addressed the histology following implant loss. Scarano et al. [33] reported that in cases of late peri-implantitis, macrophages and necrotic areas were found in the specimens examined. However, they examined only five cases involving three different materials (bovine, porcine, and phytogenic hydroxyapatite), and no other studies supported their findings. The results of this study do not align with the findings observed in the histological samples (6 of 12 cases) in this case series. Necrotic areas were detected in only one sample. Macrophages were not detected. Though the absence should be interpreted cautiously because sampling is focal and temporally remote from the original biological event. Only changes in plasma cells, lymphocytes, and fibroblasts were observed in five of the six samples. Eosinophils and neutrophils were detected in four and two cases, respectively. However, the absence of osseoconduction also provides a rationale for the predominance of two-stage implant placement in lost implants. Using a one-stage approach, the implant demonstrated sufficient primary stability to facilitate healing even in the absence of osseoconduction of the graft material. This is plausible but highly speculative because treatment allocation was not randomized and only a small number of cases are involved.

Histological findings observed in half of the patients suggested that a primary infectious cause was improbable. Other potential causes, including chronic sinusitis resulting from the overfilling of the sinus cavity or Schneiderian membrane perforation associated with implant placement [11], were excluded based on radiographic or clinical findings. Although foreign bodies were detected within the maxillary sinus in two cases due to non-compliance with postoperative instructions, this observation does not account for the observed late complications. Furthermore, no intraoperative complications during the primary procedure, such as Schneiderian membrane perforation, bleeding events, or wound dehiscence associated with impaired healing outcomes [11], were identified. Complete absence of graft osseointegration was not observed in any case. However, the incomplete bone formation noted in some patients may raise the possibility of a disturbance during the early phase of wound healing. Presently, the available evidence is insufficient to allow definitive conclusions regarding the underlying cause; thus, no definitive recommendations can be made. Additional case reports, case series, and studies involving larger patient cohorts may provide further insight into the aetiology of this complication.

### 4.3. Limitations

As previously stated, this study does not provide definitive recommendations for action. Numerous factors have contributed to this phenomenon. Except for the exceedingly small sample size, which precludes the possibility of further statistical analysis due to its marked heterogeneity, the underlying reasons for this phenomenon are human in nature. There is a risk that data may not have been recorded due to underreporting or that the physician may not have been adequately informed due to outdated medical history forms. It is imperative that patients provide accurate information to their doctors, including details of their medical conditions, current medications, and medical histories. It is not uncommon for this information to be incomplete, and even if a patient provides details to the best of their knowledge, it does not guarantee that they are aware of an undiagnosed condition, such as diabetes, at the time of the procedure. With a larger sample size, this would certainly carry less weight as the vast majority of participants provide complete information. However, since this is a case series, outliers have a much greater impact. Moreover, it is important to note that histology provides only a momentary snapshot, offering no comprehensive representation of a patient’s therapeutic trajectory. Furthermore, the study did not compare the results with those of correctly healed graft materials, which further undermines its conclusion. Moreover the retrospective design, complication-selected sampling, absence of a contemporaneous unaffected control group, uncertain/unequal follow-up of the original cohort, inability to estimate robust incidence without censoring analysis, small number of cases, patient/site heterogeneity, variation in one-stage versus two-stage treatment, several implant systems, variable graft quantity, previous grafting and local surgery, missing/retrospective compliance information, histology in only 6/12 patients, absence of microbiological testing, absence of standardized histomorphometry, absence of quantitative radiographic graft-volume analysis, inability to establish causality, overlap with a previously reported institutional cohort and possibility of referral/ascertainment bias has to be mentioned.

### 4.4. Future Research

Should the loss of bone grafts be confirmed in a larger cohort, further investigations into bovine xenohybrid bone graft materials are required. Currently, there is limited literature on this subject.

## 5. Conclusions

The observed proportion of late atypical complications was found to be 25.5%, necessitating additional cases. Even after considering the clinical, radiological, and histological findings, the aetiology remains unclear, and no definitive recommendations can be made. The available histological specimens did not demonstrate features diagnostic of an acute suppurative bacterial process; however, infection cannot be definitively excluded. Furthermore, the probability of infection from the original MSFA was considered minimal. No intra-operative complications were observed. The individual risk factors were evaluated preoperatively and managed accordingly. Available clinical records did not identify major systematic non-adherence, although postoperative behavioral compliance was not prospectively or systematically assessed. In the absence of an alternative explanation, one can hypothesize that a potential safety signal has emerged that warrants independent monitoring and comparative investigation. Possible mechanistic causes for this could include impaired early vascularization/remodeling, material-related degradation or a host reaction, mechanical effects or effects of the graft volume, subclinical sinus pathology, a mild infection/biofilm formation, patient-specific healing factors, and early clot instability. Furthermore, in theory, all patients were eligible for re-treatment after only the non-osnoconductive portion of the graft had been removed, provided they had been symptom-free for at least 6 months. In reality, however, only 3 reoperations have been performed to date.

## Figures and Tables

**Figure 1:**
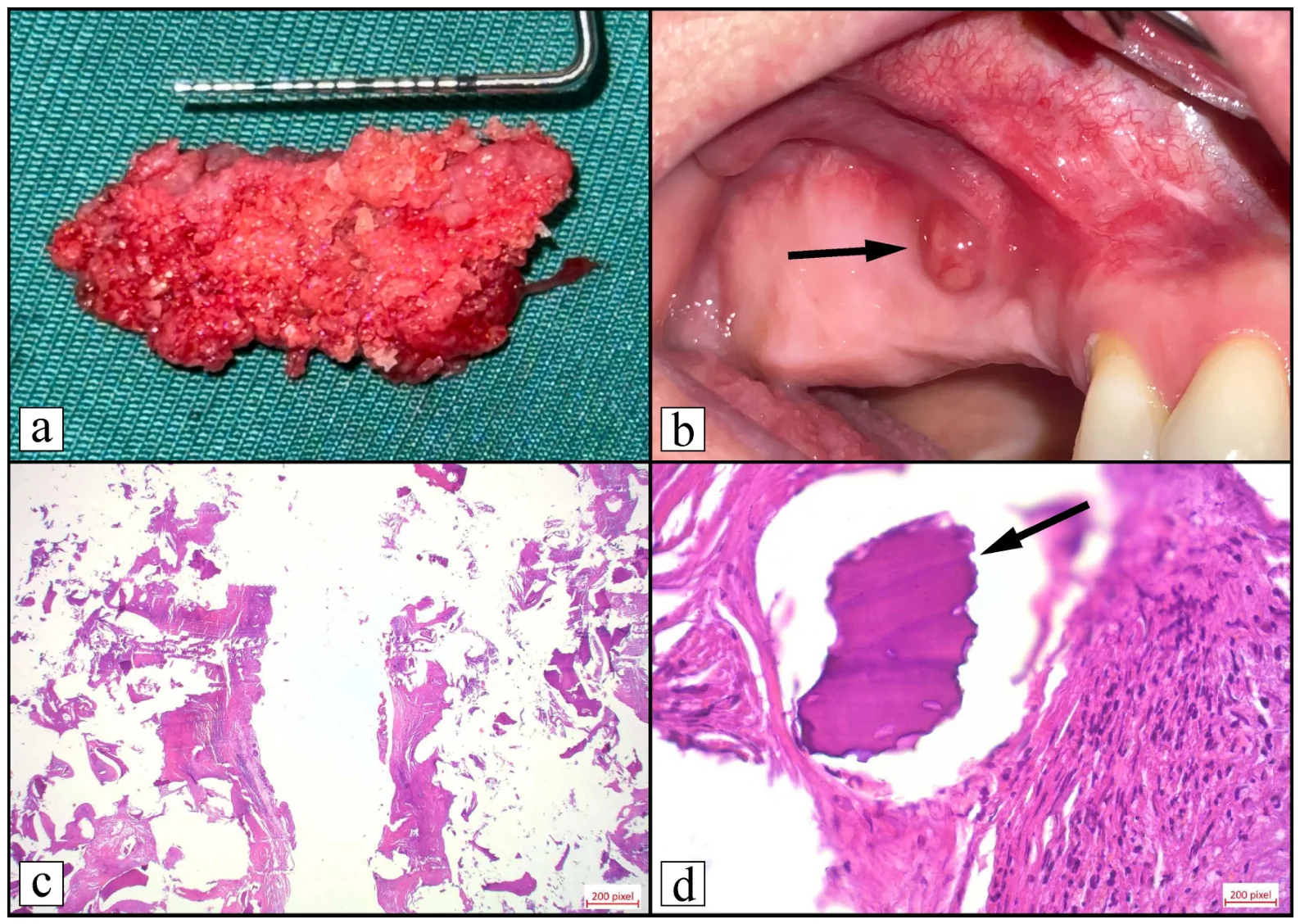
Intraoperative findings and histology: (**a**) Organized mass of the removed, non-osseointegrated CEM; small, loose CEM particles visible on the surface; (**b**) Fistula tract in the anterior region of the maxillary sinus; (**c**) Histology shows abundant inflammatory tissue; (**d**) Clear demarcation of the non-osseointegrated KEM, surrounded by inflammatory cells.

**Figure 2:**
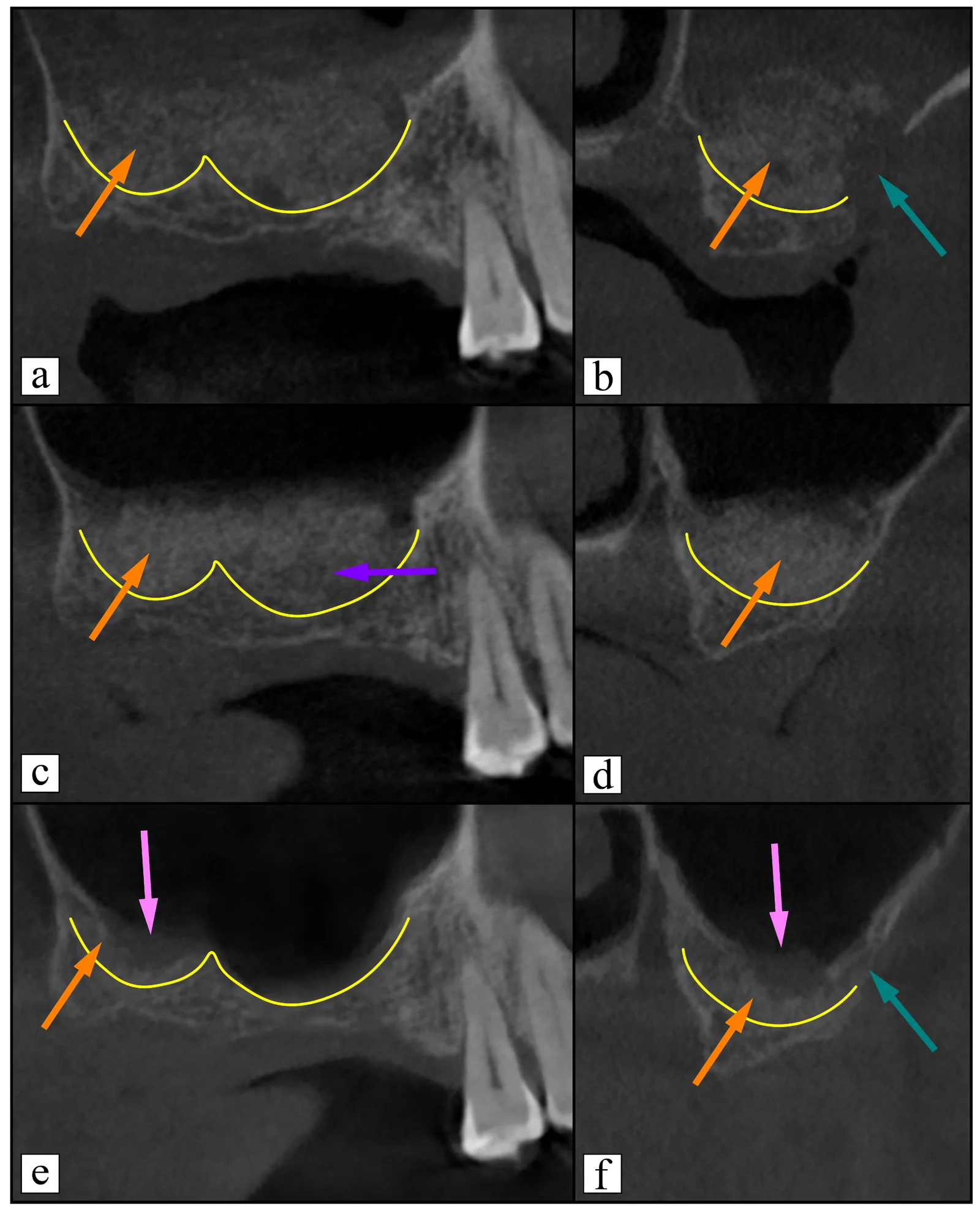
Radiographic overview of a treatment course (DVT): (**a**) Immediately postoperative view (longitudinal): sinus floor (yellow line), KEM (orange arrow); (**b**) Immediately postoperative view (frontal): sinus floor (yellow line), KEM (orange arrow), fenestration (blue arrow); (**c**) 2.7 years postoperative view (longitudinal): well-osseointegrated material distally (orange arrow), mesial inhomogeneous distribution of KEM with persistent symptoms (purple arrow); (**d**) 2.7 years postoperative view (frontal): homogeneous distribution of KEM in the distal region (orange arrow); (**e**) Current status (longitudinal): Remnants of KEM (orange arrow) with swollen subperiosteal space exclusively in the distal region (pink arrow); (**f**) Current status (frontal): Remnants of KEM (orange arrow) with swollen subperiosteal space exclusively in the distal region (pink arrow), fenestration (blue arrow).

**Table 1:** Baseline patient characteristics.

Patient	Pre-Existing Conditions and Further Risk Factors	Medications (Agent)	MSFA Region	History
A. A., ♀, 50 years	none known; ASA I	no intake	in region 25–27 (FDI) or 13–15 (UNS)	26 (FDI)/14 (UNS): atraumatic tooth extraction 27 (FDI)/15 (UNS): osteotomy alveolar ridge preservation with naturesQue SemOss B^®^ (BEGO GmbH, Bremen, Germany)
G. E., ♂, 66 years	none known; ASA I	no intake	in region 25–27 (FDI) or 13–15 (UNS)	25–27 (FDI)/13–15 (UNS): atraumatic serial extraction
G. F., ♂, 54 years	none known; ASA I	no intake	in region 16–17 (FDI) or 2–3 (UNS)	16 (FDI)/3 (UNS): surgical removal of *radix occulta in antro* 17 (FDI)/2 (UNS): atraumatic tooth extraction closure of a mouth-antral fistula
N. G., ♀, 42 years	none known; ASA I	no intake	in region 16 (FDI) or 3 (UNS)	16 (FDI)/3 (UNS): atraumatic tooth extraction cystectomy (histology: radicular cyst)
G. H., ♂, 45 years	N/A, ASA I	not specified (montekulast), Spiriva^®^ (tiotropium), Symbiocort forte Turbohaler^®^ (budesonide and formoterol fumarate dihydrate), Berodual^®^ (fenoterol hydrobromide and ipratropium bromide)	in region 26–27 (FDI) or 14–15 (UNS)	N/A
S. H., ♀, 55 years	none known; ASA I	contraceptive (not specified)	in region 14–16 and 24–26 (FDI) or 3–5 and 12–14 (UNS)	14–16 and 24–26 (FDI)/3–5 and 12–14 (UNS): edentulous upper jaw (at the time of the preliminary consultation)
F. K., ♂, 74 years	none known; ASA I	no intake	in region 24–27 (FDI) or 12–15 (UNS)	24 (FDI)/12 (UNS): atraumatic tooth extraction alveolar ridge preservation with Puros^®^ Allograft Spongiosa (ZimVie, Westminster, CO, USA) 25 (FDI)/13 (UNS): osteotomy alveolar ridge preservation with Bio-OSS^®^ (Geistlich Pharma AG, Wolhusen, Switzerland) 26 (FDI)/14 (UNS): atraumatic tooth extraction
E.-M. K., ♀, 67 years	hypertonia, information regarding increased blood loss during previous dental procedures, CMD, latex allergy, 5–10 cigarettes a day, regular alcohol intake (3–4 times a week); ASA II	N/A	in region 25–27 (FDI) or 13–15 (UNS)	25 (FDI)/13 (UNS): at the time of the preliminary consultation, the tooth was extracted 26–27 (FDI)/14–15 (UNS): osteotomy alveolar ridge preservation with naturesQue SemOss B^®^ (BEGO GmbH, Bremen, Germany)
H.-L. L., ♀, 62 years	hypertonia, thyroid dysfunction, steatotic liver disease of unclear origin, CMD, copper allergy; ASA II	Euthyrox^®^ (levothyroxine), Blopress^®^ (candesartan)	in region 14–16 (FDI) or 3–5 (UNS)	14–16 (FDI)/3–5 (UNS): atraumatic tooth extraction alveolar ridge preservation with Puros^®^ Allograft Spongiosa (ZimVie, Westminster, CO, USA)
M. S., ♂, 50 years	none known; ASA II	no intake	in region 26 (FDI) or 14 (UNS)	26 (FDI)/14 (UNS): osteotomy and cystectomy (histology: acute inflammatory follicular cyst) closure of a mouth-antral fistula
F. S., ♂, 75 years	hypertonia, type 2 diabetes mellitus; ASA II	Glucophage^®^ (metformin), Exforge HCT^®^ (amlodipine, valsartan, hydrochlorothiazide), Quetialan^®^ (quetiapin)	in region 26–27 (FDI) or 14–15 (UNS)	26–27 (FDI)/14–15 (UNS): free end situation in the second quadrant at the time of the preliminary consultation
C. V., ♀, 53 years	none known; ASA I	no intake	in region 15–17 (FDI) or 2–4 (UNS)	15 (FDI)/4 (UNS): atraumatic tooth extraction closure of a mouth-antral fistula 16–17 (FDI)/2–3 (UNS): atraumatic tooth extraction

Explanatory notes: The patients were listed in alphabetical order. FDI—Fédération Dentaire Internationale notation system. UNS—American Dental Association Universal Numbering System. CMD—cranio-mandibular-dysfunction. N/A—not available.

**Table 2:** Outcomes and follow-up.

Patients	Conditions	Symptoms & Findings	Interventions	Current Situation
A. A., ♀, 50 years	surgical: bilateral MSFA two-stage procedure involving implantation (Bego S-Line, BEGO, Bremen, Germany) 188 days after MSFA in regions 26 and 27 (FDI), 14 and 15 (UNS) one-stage procedure involving MSFA and implantation (Bego S-Line, BEGO, Bremen, Germany) in regions 15 and 16 (FDI), 3 and 4 (UNS) prosthetic: implant recovery of all four implants 419 days after the two-stage and 187 days after the one-stage procedure implant impression and manufacture of fixed, splinted crowns on both sides	The implant 27 (FDI) or 15 (UNS) has not osseointegrated 552 days after MSFA and 364 days after implantation, and shows high mobility level at the time of prosthetic restoration insertion. Following a period of 565 days since MSFA and 377 days since implantation, the patient first reported a persistent feeling of pressure, which subsequently progressed to persistent aching pain in region 26 (FDI) or 14 (UNS). After 51 days, intraoral mucosal swelling is observable, whilst the radiographic images demonstrate significant swelling of the maxillary sinus mucosa.	The removal of implants 26/27 (FDI) and 14/15 (UNS), respectively, is to be conducted, along with the complete curettage of the non-integrated augmentation material and granulation tissue. It is worthy of note that: The lateral bone window has failed to close. Subsequent X-ray imaging revealed a decrease in the mucosal swelling of the maxillary sinus. The intraoral findings were unremarkable, and the patient exhibited no symptoms. Following a period of 910 days since primary augmentation, a single-stage re-MSFA (Endobon^®^ Xenograft, ZimVie, Westminster, CO, USA) with implant placement (Biomet 3i, ZimVie, Westminster, CO, USA) was performed without complications.	No subsequent complications were observed, and the implant-supported, fixed prosthetic restoration was finally accomplished.
G. E., ♂, 66 years	surgical: unilateral MSFA two-stage procedure with implantation (Biomet 3i, ZimVie, Westminster, CO, USA) 181 days after MSFA in regions 25–26 (FDI), 13–14 (UNS) prosthetic: prosthetic rehabilitation had not yet been initiated at the time of the initial clinical symptoms	12 days after MSFA, the patient reports an episode of sneezing followed by swelling at the surgical site. Radiographic analysis has revealed the leakage of some augmentation material. The symptoms subsequently subsided. 181 days after the implant procedure, a fistula manifested in the vestibular region. A CBCT scan reveals a clear defect. The patient reports a persistent sensation of pressure.	Given the rapid improvement of symptoms 12 days after MSFA, a conservative approach was adopted. Following the formation of a fistula tract, it was excised, and the granulating inflammatory tissue was completely removed. Subsequent close monitoring revealed a swift recovery, with the patient becoming asymptomatic and the oral cavity clear of any abnormalities.	Following a period of six months during which no symptoms were observed, the patient was supplied with fixed prosthesis consisting of a three-unit free-end bridge.
G. F., ♂, 54 years	surgical: unilateral MSFA two-stage procedure with implantation (Biomet 3i, ZimVie, Westminster, CO, USA) 370 days after MSFA in regions 16–17 (FDI), 2–3 (UNS) simultaneously with the MSFA, the removal of a *radix occulta in antro* was conducted prosthetic: the prosthetic rehabilitation was completed externally	382 days after MSFA and 12 days after implantation, a tenderness-sensitive swelling became apparent. Subsequent follow-up revealed no radiological or clinical abnormalities in the control group. Following a period of 837 days since MSFA and 467 days since implantation, the patient exhibited implant mobility at 16 (FDI) and 3 (UNS), respectively. Radiographic analysis reveals advanced peri-implant tissue loss.	The implant at 16 (FDI) and 3 (UNS), respectively, was extracted, the granulation tissue was completely excised, and the resulting defect was filled with Spongostan™ (Johnson & Johnson, New Brunswick, NJ, USA). In a separate surgical procedure, it became apparent that certain components of the bone substitute material had failed to osseointegration. These sections were completely excised. The resulting defect was filled with Puros^®^ Allograft Spongiosa (ZimVie, Westminster, CO, USA) 930 days after MSFA.	A subsequent implant placement, possibly involving re-MSFA, is pending. The procedure was postponed due to the presence of an apical infection in the adjacent tooth.
N. G., ♀, 42 years	surgical: unilateral MSFA one-stage procedure with implantation (Bego S-Line, BEGO, Bremen, Germany) in region 16 (FDI), 3 (UNS) prosthetic: impression was taken and a single implant-supported crown was integrated	1047 days following an external sinus lift and implant placement, the patient exhibited a loose implant. The patient had no further complaints.	The explantation of the mobile implant was performed atraumatically, and all non-osseointegrated augmentation material, as well as connective tissue, was meticulously removed. The absence of oral-antral communication was also noted. The resulting defect was filled with Spongostan™ (Johnson & Johnson, New Brunswick, NJ, USA).	The subsequent medical examination did not reveal any clinical or radiological abnormalities. The patient has not exhibited any symptoms. The patient is awaiting further treatment.
G. H., ♂, 45 years	surgical: unilateral MSFA two-stage procedure with implantation (Bego S-Line, BEGO, Bremen, Germany) 119 days after MSFA in regions 26–27 (FDI), 14–15 (UNS) prosthetic: prosthetic rehabilitation had not yet been initiated at the time of the initial clinical symptoms	Following a period of 753 days since MSFA and 634 days since implantation, it was determined that implant 26 (FDI), 14 (UNS) had not undergone osseointegration during recovery. The patient exhibited no symptoms, and no clinical or radiological abnormalities had been detected during previous check-ups.	Following the exposure of the surgical site using a mucoperiosteal flap and the preparation of the surgical site, the implant in region 26 (FDI) or 14 (UNS) was explanted, along with all surrounding, non-osseointegrated augmentation material. The implant in region 27 (FDI) or 15 (UNS) has undergone full integration and has been fitted with a cover screw. Consequently, the clinical and radiographic findings were unremarkable. The patient reported no symptoms. 1211 days after the initial MSFA, 1092 days after implantation and 458 days after explantation and removal of the non-osseointegrated graft material, a re-MSFA was performed using Endobon^®^ Xenograft (ZimVie, Westminster, CO, USA).	21 days after re-MSFA, the patient exhibited symptoms, characterised by a dull sensation and swelling. The patient reports having blown his nose and sneezed with his mouth closed on several occasions over the past few days, in contravention of the post-operative instructions. Radiographic examination revealed the presence of a fistula tract and an augment within the sinus lumen. The treatment plan was devised with the objective of administering antibiotics and adopting a wait-and-see approach. The patient is currently asymptomatic.
S. H., ♀, 55 years	surgical: bilateral MSFA two-stage procedure with MSFA in regions 14–16 and 24–26 (FDI), 3–5 and 12–14 (UNS) in addition to the MSFA, individualised graft blocks (**Name, Hersteller**) were fixed bilaterally within onlay grafting procedure 288 days after augmentation (MSFA + onlay), implantation (Straumann BLX SL-Active, Institut Straumann AG, Basel, Switzerland) in regions 11, 13, 15, 21, 23, 25 (FDI) and 4, 6, 8, 9, 11, 13 (UNS), respectively during the implantation procedure, bone augmentation was performed distally from implant 25 (FDI) or 13 (UNS) using Puros^®^ Allograft Spongiosa (ZimVie, Westminster, CO, USA) prosthetic: prosthetic rehabilitation with implant-supported bridges	1593 days after augmentation (onlay + MSFA), 1315 days after implantation and 713 days after prosthetic restoration, the patient presented with mild but persistent discomfort in the right MSFA region. The facial wall of the maxillary sinus exhibits tenderness to pressure, and upon exterior observation, the cheek demonstrates slight swelling. A thickening of the Schneiderian membrane is visible on the DVT scan; however, there is no evidence of implant or bone substitute material abnormalities.	A watch-and-wait approach is recommended, with frequent follow-up examinations. Ten days following the initial onset, the clinical picture remains unchanged.	The patient is expected to attend subsequent check-ups and follow-up appointments at regular intervals. The patient’s current symptoms and clinical findings do not, at present, warrant an invasive procedure.
F. K., ♂, 74 years	surgical: unilateral MSFA two-stage procedure with implantation (Biomet 3i, ZimVie, Westminster, CO, USA) 176 days after MSFA in regions 24 and 26 (FDI), 12 and 14 (UNS) prosthetic: prosthetic rehabilitation had not yet been initiated at the time of the initial clinical symptoms	Following a period of 386 days since MSFA and a period of 210 days since implantation, it was established that implant 26 (FDI) or 14 (UNS) had not undergone osseointegration, a finding made evident during the process of taking the impression. The patient had no prior symptoms, and the follow-up examinations revealed no clinical abnormalities.	The affected implant was explanted, and all non-osseointegrated augmentation material was curetted out. The maxillary sinus mucosa demonstrates polypoid changes with an abnormal yellowish discolouration (**histology: ???**). The implant and peri-implant tissues were unremarkable at 24 (FDI) or 12 (UNS), so this implant was fitted with a prosthetic, single-crown restoration. The patient was subsequently symptom-free, and the clinical and radiological findings were unremarkable. 737 days after the initial MSFA and 351 days after explantation and removal of the non-osseointegrated material, the re-MSFA was performed in a single-stage procedure involving implantation (Biomet 3i, ZimVie, Westminster, CO, USA) and augmentation (Endobon^®^ Xenograft, ZimVie, Westminster, CO, USA).	In consideration of the healing phase, the implants were restored in a collective configuration as part of an implant-supported bridge construction. The patient has undergone prosthetic rehabilitation and is now free of symptoms.
E.-M. K., ♀, 67 years	surgical: unilateral MSFA one-stage procedure with implantation (Biomet 3i, ZimVie, Westminster, CO, USA) in regions 25–27 (FDI), 13–15 (UNS) 148 days after MSFA, extraction of tooth 24 (FDI) or 12 (UNS) and alveolar ridge preservation (**Material?**) 168 days after extraction of 24 (FDI) or 12 (UNS), implantation (Biomet 3i, ZimVie, Westminster, CO, USA) prosthetic: the implants were restored with a long-term temporary bridge restoration based on PMMA (**warum?**)	The patient presented 645 days after MSFA with a sensation of swelling in the second quadrant. A thorough clinical and radiological examination did not reveal any abnormalities. The symptoms exhibited a persistent nature throughout the documented observation period, which extended for a duration of 259 days.	In the absence of any clinical or radiographic abnormalities, and at the patient’s express demand, the long-term provisional PMMA bridge was remodelled in the laboratory on five occasions during the period in question, without any sign of an improvement in symptoms.	The patient has ceased treatment and is now undergoing treatment elsewhere.
H.-L. L., ♀, 62 years	surgical: unilateral MSFA one-stage procedure with implantation (Bego S-Line, BEGO, Bremen, Germany) in regions 14–16 (FDI), 3–5 (UNS) prosthetic: prosthetic rehabilitation was conducted externally	1003 days after MSFA, the patient presented with lasting symptoms. The clinical examination revealed no abnormalities. 14 days later, the patient presented again with persistent, pulling symptoms. Upon intraoral examination, the region 16–17 (FDI) or 2–3 (UNS) is found to be tender to pressure. Radiographic analysis reveals the presence of vertical bone defects in regions 14–16 (FDI) or 3–5 (UNS).	The patient has cancelled the previously agreed appointment, which was scheduled to discuss the next steps.	The patient is currently undergoing further treatment externally.
M. S., ♂, 50 years	surgical: unilateral MSFA one-stage procedure with implantation (Bego S-Line, BEGO, Bremen, Germany) in region 16 (FDI), 3 (UNS) prosthetic: prosthetic rehabilitation was conducted externally	600 days after MSFA, the patient presented with implant loss following a prosthetic rehabilitation outside the department. Radiographs reveal a large bone defect. The patient reports no discomfort.	735 days after the initial MSFA, the procedure involving re-MSFA was planned. Intraoperatively, the integrity of the facial wall of the maxillary sinus was confirmed. It was evident that the explantation site had undergone osseous closure. The situation permitted conventional implantation to be carried out adjacent to the original implantation and augmentation site, obviating the necessity for further measures (Biomet 3i, ZimVie, Westminster, CO, USA).	The newly placed implant was successfully fitted with a crown, with no further complications arising.
F. S., ♂, 75 years	surgical: unilateral MSFA two-stage procedure with implantation (Biomet 3i, ZimVie, Westminster, CO, USA) 196 days after MSFA in regions 26–27 (FDI), 14–15 (UNS) prosthetic: prosthetic rehabilitation had not yet been initiated at the time of the initial clinical symptoms	Subsequent to the exposure of the implants, it was observed during the impression-taking process that neither implant had osseointegrated. The patient had not exhibited any symptoms prior to this point in time. At this stage, no abnormalities were observed in the oral cavity. Subsequent to this, the patient exhibited signs of discomfort upon pressure application.	Following a period of 769 days since MSFA and 573 days since implantation, the implants were explanted, and the peri-implant, non-integrated graft material was removed. At that time, a positive OAC was identified. Due to the persistence of the condition, accompanied by sporadic discharge (non-purulent) and radiographically evident swelling of the maxillary sinus mucosa, a further surgical procedure was performed. The procedure entailed an extended curettage, followed by multi-layered wound closure utilising a Bichat fat pad.	The patient is currently asymptomatic. The available clinical and radiological findings suggest that the wound is healing in accordance with the expected norm. The intention is to perform a re-MSFA.
C. V., ♀, 53 years	surgical: unilateral MSFA MSFA procedure in regions 15–17 (FDI) or 2–4 (UNS) prosthetic: implantation or prosthetic rehabilitation had not yet been initiated at the time of the initial clinical symptoms	Following an external sinus lift procedure, which occurred 488 days previously, the patient exhibited a fistula tract on the vestibular side of the MSFA region, accompanied by displaced particles of the bone graft material. Intraorally, the mucosa is visibly swollen. Following a period of asymptomaticity, the patient presented with recurrent blistering and leakage of augmentation material 943 days after MSFA.	Intraoperatively, a fistulous tract in the facial maxillary sinus wall was confirmed. The bone graft was not osseointegrated in the affected area. It was totally removed. The patient subsequently reported severe pain and rhinorrhea, which resulted in a referral to an otolaryngologist. The consultation revealed that the condition had largely normalised, with the fistula tract having healed completely.	1370 days after MSFA, the patient is symptom-free, and the radiological and clinical findings are unremarkable. The intention is to undertake a re-MSFA with single-stage implantation.

Explanatory notes: The patients were listed in alphabetical order. Abbreviations: MSFA—maxillary sinus floor augmentation. FDI—Fédération Dentaire Internationale notation system. UNS—American Dental Association Universal Numbering System. CBCT—Cone Beam Computed Tomography. PMMA—polymethyl methacrylate. OAC—oroantral communication.

**Table 3:** Standardised outcome measures.

Outcome Measures	Assessment Method	Patients											
		A. A.	G.E.	G.F.	N.G.	G.H.	S.H.	F.K.	E.-M.K. ^֍^	H.-L.L. ^֍^	M.S.	F.S.	C.V.
**1.** **clinical outcomes: intraoperative or early healing complications**													
sinus membrane perforation	clinical examination	-	-	-	-	-	-	-	-	-	-	-	-
haemorrhage-related complication	clinical examination	-	-	-	-	-	-	-	-	-	-	-	-
oroantral communication	Valsalva maneuver, Bowman probe	-	-	-	-	-	-	-	-	-	-	-	-
sinusitis symptoms: dull, pressing sensation, nasal secretion, obstruction, …	clinical examination	-	• *	-	-	• **	-	-	-	-	-	-	-
postoperative infection signs: e.g., secretion, suppuration, redness, fever, swelling, …	clinical examination	-	• *	• *	-	-	-	-	-	-	-	-	-
pain/symptoms/disorders/discomfort	interview	-	• *	-	-	• **	-	-	-	-	-	-	-
wound dehiscence	clinical examination	-	-	-	-	-	-	-	-	-	-	-	-
**2.** **clinical outcomes: late complications**													
fistula tract (intraoral or extraoral)	clinical examination	-	• *	-	-	• **	-	-	-	-	-	-	• *
sinusitis symptoms: dull, pressing sensation, nasal secretion, obstruction, …	clinical examination	-	-	-	-	-	• *	• *	-	• *	-	•*	• *
other signs of infection: e.g., secretion, suppuration, redness, fever, swelling, …	clinical examination	• *	-	-	-	-	• *	-	• *	• *	-	-	-
oroantral communication	Valsalva maneuver, Bowman probe	-	-	-	-	-	-	-	-	-	-	• *	-
pain/symptoms/disorders/discomfort	interview	• *	• *	-	-	-	• *	-	• *	• *	-	• *	-
partial or total loss of augmentation	clinical examination	• *	-	• *	• *	• *	-	• *	-	-	-	• *	• *
implant loss	clinical examination	• *	-	• *	• *	• *	-	• *	-	-	• *	• *	-
**3.** **radiological outcomes**													
sinus opacification or maxillary sinus mucosa swelling	OPG, CBCT	• *	-	-	-	-	• *	-	-	-	-	-	• *
loss of peri-implant augmentation volume (loss of graft-implant-contact)	CBCT	• *	-	• *	-	-	-	-	-	• *	• *	-	-
radiographic signs of failed osseointegration	CBCT	-	-	-	-	• *	-	-	-	-	-	-	-
location of graft material inside the sinus	OPG, CBCT	-	• *	-	-	• **	-	-	-	-	-	-	• *
**4.** **functional outcomes**													
delay in prosthetic rehabilitation	-	• *	• *	• *	• *	• *	-	• *	N/A	N/A	• *	• *	• *
failure to provide a prosthetic rehabilitation	-	• *	-	-	-	• *	-	-	N/A	N/A	-	• *	-
prosthetic loss	-	-	-	• *	• *	-	-	-	N/A	N/A	• *	-	-
currently without prosthetic restoration	-	-	-	• *	• *	• **	-	-	N/A	N/A	-	• *	• *

Explanatory notes: • *—during or in association with primary MSFA. • **—during or in association with re-MSFA. ֍—no longer a patient at the department. N/A—no information.

## Data Availability

The original contributions presented in this study are included in the article. Further inquiries can be directed to the corresponding author.
